# Long-lasting effect of oral azithromycin taken by women during labour on infant nutrition: Follow-up cohort of a randomized clinical trial in western Gambia

**DOI:** 10.1371/journal.pone.0206348

**Published:** 2018-10-25

**Authors:** Anna Roca, Bully Camara, Claire Oluwalana, Kodou Lette, Christian Bottomley, Umberto D’Alessandro

**Affiliations:** 1 MRC Unit at the London School of Hygiene and Tropical Medicine, Fajara, The Gambia; 2 London School of Hygiene and Tropical Medicine, London, United Kingdom; Public Library of Science, UNITED KINGDOM

## Abstract

**Objective:**

To assess the effect of administering an oral dose of 2g of azithromycin in Gambian women during labour on infant growth.

**Methods:**

Children whose mothers had been randomized to receive either an oral dose of 2g of azithromycin or placebo during labour were visited at home at the end of infancy by trained study nurses blind to the treatment allocation. The follow-up visit of these cohorts (exposed and non-exposed to azithromycin), which was not part of the original trial design, was conducted between November 2014 and May 2015 when the infants were 11 to 13 months of age. During visits, nurses recorded anthropometrical measurements and transcribed information from the infants’ welfare cards.

**Results:**

Four-hundred and sixty-five (79.6%) of the 584 infants aged 11–13 months at the time of the survey were recruited. The proportion of children with an age-adjusted Z-score <-2SD for mid-upper-arm circumference (MUAC) was lower among those exposed to azithromycin [1.3% versus 6.3%, OR = 0.21 95%CI (0.06,0.72), p = 0.006] and there was weak evidence of a difference in the proportion of infants with weight-for-age (WAZ) Z-score <-2SD [7.1% versus 12.1%, OR = 0.58 95%CI (0.33,1.04), p = 0.065]. For all other malnutrition indicators the proportions were similar in the exposed and un-exposed cohort.

**Conclusions:**

Our results show that azithromycin in labour may have a beneficial effect in MUAC among children who are below the curve. Larger studies with closer follow-up are warranted.

**Trial registration (main trial):**

ClinicalTrials.gov Identifier NCT01800942.

## Introduction

Azithromycin is a broad spectrum antibiotic used to treat infectious diseases such as pneumonia, middle ear infections, chlamydia, gonorrhoea and malaria. It has also been used in mass drug administration (MDA) campaigns to control trachoma [1]. Such campaigns have reported beneficial effects beyond the target disease. In Ethiopia, MDA with azithromycin for trachoma control reduced all-cause mortality in 1–9 years old children by 49% [2] and in The Gambia MDA reduced asymptomatic pneumococcal carriage [3]. While the mechanism underlying the mortality reduction reported in Ethiopia was not clear, *Porco et al* suggested that it may have been due to an improvement in nutritional status among children who received azithromycin [2]. A subsequent systematic review of the association between antibiotic use and growth found that antibiotic use promotes linear growth and weight gain in children in low and middle-income countries [4], where the prevalence of malnutrition in children is high. The hypothesis is also supported by data from randomized clinical trials using azithromycin and anti-malarial drugs during pregnancy, which have shown that this combination increases birth weight [5–7] and reduced the incidence of stunting in childhood [8].

We recently conducted a trial in Western Gambia in which women in labour were randomized to receive oral azithromycin or placebo [9]. The primary outcome of the trial was neonatal nasopharyngeal carriage of *Staphylococcus aureus*, Group B Streptococcus or *Streptococcus pneumoniae*. Maternal bacterial carriage in the nasopharynx, breast milk and low vaginal tract were also assessed. Prevalence of carriage of study bacteria was lower in the first month after birth in mothers who had received azithromycin and their newborns [10]. In addition, fewer episodes of maternal and neonatal illness were reported in the azithromycin arm during the 8 weeks of follow-up [11].

We conducted a follow up visit of children at 1 year of age, to compare child growth between those who were exposed to azithromycin (because the mother received azithromycin during labour) and those who were not exposed. Our hypothesis was that by reducing bacterial carriage during early life, azithromycin would reduce malnutrition, a major risk factor for child mortality [12].

## Methods

### Study site and population

The study was based at the Jammeh Foundation for Peace (JFP), a government-run health centre located in Western Gambia that manages 4,500 deliveries per year [10]. The population covers the main ethnic groups in The Gambia and illiteracy is high. The climate of the area is typical of the sub-Sahel region.

### Main trial

The study protocol has been described elsewhere [9]. Briefly, this was a phase-III, double-blind, placebo-controlled trial in which 829 pregnant women attending the labour ward in JFP were randomized to receive a single oral dose of 2g of either oral azithromycin or placebo (ratio 1:1). The primary endpoint of the trial was prevalence of *S*. *aureus*, GBS or *S*. *pneumoniae* carriage in the NPS sample of the newborn at day 6 and secondary endpoints included disease and hospitalization in women and newborns during the 8 weeks of follow up. Secondary analysis included estimating the effect of the intervention on clinical disease during the 8 weeks of follow-up [11]. The trial started in April 2013 and lasted 14 months.

The packaging and labeling of the investigational medicinal product (IMP) was conducted byIdifarma. Azithromycin and placebo were provided as tablets packed in blisters. One blister pack of IMP contained 4 tablets of 0.5g (2g) of either azithromycin or placebo. The randomisation list was created by an independent data manager and Idifarma numbered the blisters according to the list [9]. The active drug and the placebo looked identical.

### Follow-up visit during infancy

Between November 2014 and May 2015, a follow-up visit at home was conducted for children aged 11–13 months whose mothers had been randomized to receive either an oral dose of 2g of azithromycin (exposed) or placebo (non-exposed). These children were visited at home by a trained nurse blind to the treatment allocation and anthropometric measurements were recorded. Children were weighed on a standard calibrated scale (Tanita scale) which measures to a precision of 0.1kg. Height was measured using a standard calibrated length board with a precision of 0.1cm. We used a standard coloured and gradated tape to measure mid-upper-arm circumference (MUAC) and a suitable measuring tape for the head circumference, both with a precision of 0.1cm. During the visits, the nurse also transcribed height and weight measurements from the infant’s health card onto the study questionnaire.

### Ethical approval

Both the main trial and the adhoc follow up visit were approved by the Joint MRC/Gambia Government Ethics Committee. Mothers of children participating in the survey signed an additional informed consent.

### Statistical analysis

Study questionnaires were reviewed before being double entered into *OpenClinica* (www.openclinica.com). We used WHO child-growth standards to calculate Z-scores for height-for-age (HAZ), weight-for-age (WAZ), weight-for-height (WHZ), body mass index-for-age, head circumference-for-age, and MUAC for age [WHO Anthro version 3.2.2, January 2011]. Children with z-scores <-2SD and <-3SD were classified as malnourished and severely-malnourished, respectively [13]. For each score, we used risk ratios and Fisher’s exact test to compare the proportion with a Z-score<-2 SD and <-3SD between the study arms. In addition, we used weight measurements transcribed from the welfare-card and measurements taken at 12 months to estimate growth curves (0–12 months) for children in each trial arm. The curves were estimated by fitting a spline function to the weight data using random effects linear regression. No other covariates were included in the regression. The standardized residuals obtained from fitting these models were compared between arms (residuals were standardized by dividing by the standard deviation of the residuals). Specifically, we compared the proportion of standardized residuals<-2 SD using logistic regression, with cluster-robust variance estimates. We used a p-value cut-off of 0.05 to define statistical significance. Analyses were done using Stata version 14.1.

## Results

### Study profile

In the trial, 829 women were recruited during labour, and 830 children were born alive, of whom 814 survived until the end of the 8-week follow-up period. Out of 814 children alive at 8-weeks, 5 had died and 196 were >13 months at the time of the survey. Among the 613 study children ≤13 months of age at the time of starting the long-term survey, 465 (76.0% of 613 and 57% of the 814) participated in the follow up visit (S1 Fig). Characteristics of study participants and non-participants are shown in Table 1.

**Table 1:** Characteristics of study participants who participated and not in the long term follow-up visit.

*Variables*	Categories	12 months survey included (N = 465) n/N (%)	12 months survey not included (N = 378) n (%)	p-value
Sex	Female	230 (49.5)	175 (46.3)	0.360
	Male	235 (50.5)	203 (53.7)	
Ethnicity	Mandinka	201 (43.5)	151 (41.8)	0.367
	Wollof	49 (10.6)	45 (12.5)	
	Jola	80 (17.3)	47 (13.0)	
	Fula	76 (16.5)	68 (18.8)	
	Other	56 (12.1)	50 (13.9)	
Maternal schooling	<1 year	231 (49.9)	193 (53.8)	0.271
	≥ 1 year	232 (50.1)	166 (46.2)	
Maternal age	18–19 years	30 (6.5)	37 (9.8)	0.109
	20–29 years	295 (63.4)	244 (64.6)	
	≥30 years	140 (30.1)	97 (25.7)	
Multiple pregnancy	No	453 (97.4)	362 (95.8)	0.183
	Yes	12 (2.6)	16 (4.2)	
Low birth weight (<2.5kg)	No	432 (93.1)	354 (93.9)	0.642
	Yes	32 (6.9)	23 (6.1)	

Baseline characteristics (age at follow up, gender, ethnic group, season at birth, birth weight and maternal age) of children in the azithromycin (n = 226) and the placebo group (n = 239) were similar, except for the higher number of multiple pregnancies in the placebo group (p = 0.037) (Table 2).

**Table 2:** Characteristics of study participants at birth.

*Characteristics of study children at birth*		Azithromycin (N = 226) n (%)	Placebo (N = 239) n (%)	p-value
Age at follow up (days)	Mean (SD)	372.5 (18.4)	373.6(17.7)	0.486
Mother's age (years)	Mean (SD)	26.5(5.3)	26.6(4.9)	0.684
Birth weight (kg)	Mean (SD)	3.1(0.5)	3.1(0.5)	0.172
Sex	Female	118 (52.2)	112 (46.9)	0.266
	Male	108 (47.8)	127 (53.1)	
Ethnicity	Madinka	92 (40.7)	109 (45.6)	0.490
	Jola	45 (19.9)	35 (14.6)	
	Wollof	25 (11.1)	24 (10.0)	
	Other	64 (28.3)	71 (29.7)	
Apgar score (1 minute)^2^	5–6	5 (2.2)	2 (0.8)	0.232
	7–8	16 (7.1)	10 (4.2)	
	9–10	204 (90.7)	224 (94.9)	
Multiple pregnancy	No	224 (99.1)	229 (95.8)	0.037
	Yes	2 (0.9)	10 (4.2)	
Season of birth^1^	Dry	178 (78.8)	190 (79.5)	0.909
	Rainy	48 (21.2)	49 (20.5)	

^1^Rainy season June- October; Dry season November—May

^2^ n = 1 missing Apgar score in azithromycin arm and n = 3 missing score in placebo arm

### Growth curves

The growth curves were estimated from n = 1,575 measurements in the placebo group (6.6 per child) and 1,472 measurements (6.5 per child) in the placebo group. The curves were similar for both cohorts of children (S2 Fig). The proportion of standardized residuals <-2SD was also similar in both groups [1.8% versus 3.1%, adjusted OR = 0.59 95% CI (0.22,1.59)].

### 12 months follow-up visit

The proportion with MUAC-for-age<-2SD was significantly lower in the azithromycin group [1.3% versus 6.3%, RR = 0.21 95% CI (0.06,0.72)] (Table 3). For all other anthropometrical measures the proportions were similar in both groups.

**Table 3:** Anthropometrical measurements for nutritional status by study group at the follow-up visit.

***Mean (SD)***	**Azithromycin (N = 226)** **Mean (SD)**	**Placebo** **(N = 239)** **Mean (SD)**			
*Age at follow-up (days)*	372.47(18.39)	373.64(17.70)			
Height (cm)	71.7(2.8)	71.7(3.0)			
Weight (kg)	8.8(1.1)	8.8(1.2)			
Head circumference (cm)	45.5(1.5)	45.4(1.6)			
MUAC (cm)	14.4(1.1)	14.3(1.3)			
***Z-score <-2SD*** ***N (%)***	**Azithromycin (N = 226)** **n (%)**	**Placebo** **(N = 239)** **n (%)**	**RR(95%CI)**		**p-value**
WAZ^1^ <-2SD	16(7.1)	29(12.1)	0.58(0.33,1.04)		0.065
HAZ^2^ <-2SD	58(25.7)	76(31.8)	0.81(0.60,1.08)		0.144
BMI^3^-for-age <-2SD	3(1.3)	8(3.3)	0.40(0.11,1.48)		0.152
WHZ^4^ <-2SD	5(2.2)	8(3.3)	0.66(0.22,1.99)		0.458
MUAC^5^-for-age <-2SD	3(1.3)	15(6.3)	0.21(0.06,0.72)		0.006
Head circumference-for-age <-2SD	9(4.0)	9(3.8)	1.06(0.43,2.62)		0.904
***Z-score <-3SD*** ***N (%)***	**Azithromycin (N = 226)** **n (%)**	**Placebo** **(N = 239)** **n (%)**	**RR(95%CI)**		**p-value**
WAZ^1^ <-3SD	3(1.3)	5(2.1)	0.63(0.15,2.62)		0.526
HAZ^2^ <-3SD	14(6.2)	18(7.5)	0.82(0.42,1.61)		0.569
BMI^3^-for-age <-3SD	0(0.0)	2(0.8)	0.00(NA,NA)		0.168
WHZ^4^ <-3SD	0(0.0)	2(0.8)	0.00(NA,NA)		0.168
MUAC^5^-for-age <-3SD	1(0.4)	2(0.8)	0.53(0.05,5.79)		0.596
Head circumference-for-age <-3SD	1(0.4)	3(1.3)	0.35(0.04,3.36)		0.343

^1^WAZ = Weight for age

^2^HAZ = Height for age

^3^BMI = Body mass index

^4^WHZ = Weight for height

^5^MUAC = mid-upper-arm circumference

Proxy signs for nutritional status were similar between groups (Table 4).

**Table 4:** Proxy signs for nutritional status by study arm.

*Signs for Nutritional status*	Azithromycin (N = 224) n (%)	Placebo (N = 239) n (%)	RR (95%CI)	p-value
Peeling skin lesions	0(0)	0 (0)	NA^1^	1
Bilateral pedal oedema	0 (0)	0 (0)	NA^1^	1
Sparse hair distribution	11(4.9)	18(7.5)	0.65(0.31,1.35)	0.257
Loosely fitting clothing	4(1.8)	10(4.2)	0.43(0.14,1.34)	0.176
Loose or sparse hair distribution	15(6.7)	24(10.0)	0.67(0.36,1.24)	0.241

^1^NA = Not applicable

Between the end of the trial (8 weeks) and the follow up home visit, 3 children in the azithromycin and 7 children in the placebo groups had records of hospitalization.

## Discussion

Children born to mothers treated with oral azithromycin during labour were less likely to have MUAC standardized z-score <2SD at around 1 year of age, although the mean MUAC was similar in the two study groups. It is also encouraging that the proportion of undernourished infants (<2SD for WAZ, HAZ, BMI, WHZ and MUAC) appears to be lower in the azithromycin arm, but these findings are not conclusive because only the difference in MUAC was statistically significant.

Although azithromycin MDA has been shown to be associated with decreased child mortality in Ethiopia [2], previous studies have not shown an association with nutritional status [14]. However, in the previous studies, azithromycin treatment was part of the Trachoma control strategy and was given as a single dose (either once or annually) mainly to children above 1 year of age. In contrast, in our study, although azithromycin was given at a single dose to study women during labour, it reached the infancy through the breast milk for at least 4 weeks [15]. In Malawi, adding azithromycin to the standard intermittent preventive treatment for malaria control during pregnancy showed a reduction of childhood stunting [8].

Our intervention might have reduced the incidence of malnutrition by reducing chronic gut inflammation which is common among African children. Alternatively it may have mediated changes in the intestinal microbiome which persisted beyond the trial period. Our results are in line with a systematic review which found that antibiotic use promotes linear growth and weight gain in children in low and middle-income countries [4]. Because the azithromycin arm in our study had lower bacterial carriage, our results are also consistent with the results of a previous observational study conducted in India, which found that bacterial nasopharyngeal carriage at the age of two months was associated with increased risk of malnutrition at 6 months [16].

Few children in our study had severe malnutrition (Z-scores<-3) and none had clinical signs of severe malnutrition (peeling skin lesions or bilateral pedal oedema), probably reflecting the good care that these children received during the trial follow-up. This is consistent with the low mortality observed in the study between 2–12 months of age (1/3 of that expected in The Gambia) [17], and with the low rate of hospitalization.

The study had a number of limitations. First, it was not powered to detect a difference in malnutrition. Second, approximately 20% of participants from the main trial were older than 13 months at the time of the long term CSS and were not included. The reason for this decision was that growth is strongly age-dependent and, as such, growth statistics are hard to interpret unless they are restricted to a narrow age range. Third, we did not have the resources for additional visits and were therefore unable to take further anthropometric measurements, or collect data on other important variables such as breast feeding practices. Instead we relied on weight measurements recorded on the infant’s welfare card, which were sometimes missing, to calculate growth curves.

## Conclusions

Our results suggest a possible beneficial effect of giving azithromycin during labour on the risk of infant malnutrition. However, our findings are not conclusive and may be attributable to chance. Larger studies with closer follow-up are warranted to confirm whether the intervention has any effect on nutritional status of the offspring beyond the neonatal period. These studies should record the prevalence of antibiotic resistance as a potential drawback of the intervention [10].

## Supporting information

S1 checklist Strobe check list. (PDF)

S1 Fig Study profile. (TIF)

S2 Fig Standardized residuals for growth curve during infancy according to the children welfare cards in the azithromycin and placebo groups. (TIF)

## References

[1] Harding-Esch EM, Edwards T, Mkocha H, Munoz B, Holland MJ, Burr SE, Sillah A, Gaydos CA, Stare D, Mabey DC, Bailey RL, West SK (2010) Trachoma prevalence and associated risk factors in the gambia and Tanzania: baseline results of a cluster randomised controlled trial. PLoS Negl Trop Dis 4: e861 10.1371/journal.pntd.0000861 21072224 PMC2970530

[2] Porco TC, Gebre T, Ayele B, House J, Keenan J, Zhou Z, Hong KC, Stoller N, Ray KJ, Emerson P, Gaynor BD, Lietman TM (2009) Effect of mass distribution of azithromycin for trachoma control on overall mortality in Ethiopian children: a randomized trial. JAMA 302: 962–968. 302/9/962 [pii]; 10.1001/jama.2009.1266 19724043

[3] Burr SE, Milne S, Jafali J, Bojang E, Rajasekhar M, Hart J, Harding-Esch EM, Holland MJ, Mabey DC, Sillah A, Bailey RL, Roca A (2014) Mass administration of azithromycin and Streptococcus pneumoniae carriage: cross-sectional surveys in the Gambia. Bull World Health Organ 92: 490–498. 10.2471/BLT.13.133462 BLT.13.133462 [pii]. 25110374 PMC4121870

[4] Gough EK, Moodie EE, Prendergast AJ, Johnson SM, Humphrey JH, Stoltzfus RJ, Walker AS, Trehan I, Gibb DM, Goto R, Tahan S, de Morais MB, Manges AR (2014) The impact of antibiotics on growth in children in low and middle income countries: systematic review and meta-analysis of randomised controlled trials. BMJ 348: g2267 10.1136/bmj.g2267 24735883 PMC3988318

[5] Luntamo M, Kulmala T, Cheung YB, Maleta K, Ashorn P (2013) The effect of antenatal monthly sulphadoxine-pyrimethamine, alone or with azithromycin, on foetal and neonatal growth faltering in Malawi: a randomised controlled trial. Trop Med Int Health 18: 386–397. 10.1111/tmi.12074 23432801

[6] Unger HW, Ome-Kaius M, Wangnapi RA, Umbers AJ, Hanieh S, Suen CS, Robinson LJ, Rosanas-Urgell A, Wapling J, Lufele E, Kongs C, Samol P, Sui D, Singirok D, Bardaji A, Schofield L, Menendez C, Betuela I, Siba P, Mueller I, Rogerson SJ (2015) Sulphadoxine-pyrimethamine plus azithromycin for the prevention of low birthweight in Papua New Guinea: a randomised controlled trial. BMC Med 13: 9 10.1186/s12916-014-0258-3 s12916-014-0258-3 [pii]. 25591391 PMC4305224

[7] Unger HW, Wangnapi RA, Ome-Kaius M, Boeuf P, Karl S, Mueller I, Rogerson SJ (2016) Azithromycin-containing intermittent preventive treatment in pregnancy affects gestational weight gain, an important predictor of birthweight in Papua New Guinea—an exploratory analysis. Matern Child Nutr 12: 699–712. 10.1111/mcn.12215 26373537 PMC6860090

[8] Hallamaa L, Cheung YB, Maleta K, Luntamo M, Ashorn U, Gladstone M, Kulmala T, Mangani C, Ashorn P (2018) Child Health Outcomes After Presumptive Infection Treatment in Pregnant Women: A Randomized Trial. Pediatrics. peds.2017–2459 [pii]; 10.1542/peds.2017-2459 29472491

[9] Roca A, Oluwalana C, Camara B, Bojang A, Burr S, Davis TM, Bailey R, Kampmann B, Mueller J, Bottomley C, D'Alessandro U (2015) Prevention of bacterial infections in the newborn by pre-delivery administration of azithromycin: Study protocol of a randomized efficacy trial. BMC Pregnancy Childbirth 15: 302 10.1186/s12884-015-0737-3 [pii]. 26585192 PMC4653934

[10] Roca A, Oluwalana C, Bojang A, Camara B, Kampmann B, Bailey R, Demba A, Bottomley C, D'Alessandro U (2016) Oral azithromycin given during labour decreases bacterial carriage in the mothers and their offspring: a double-blind randomized trial. Clin Microbiol Infect 22: 565–569. S1198-743X(16)30020-9 [pii]; 10.1016/j.cmi.2016.03.005 27026482 PMC4936760

[11] Oluwalana C, Camara B, Bottomley C, Goodier S, Bojang A, Kampmann B, Ceesay S, D'Alessandro U, Roca A (2017) Azithromycin in Labor Lowers Clinical Infections in Mothers and Newborns: A Double-Blind Trial. Pediatrics 139 peds.2016-2281 [pii]; 10.1542/peds.2016-2281 28130432

[12] Lazzerini M, Seward N, Lufesi N, Banda R, Sinyeka S, Masache G, Nambiar B, Makwenda C, Costello A, McCollum ED, Colbourn T (2016) Mortality and its risk factors in Malawian children admitted to hospital with clinical pneumonia, 2001–12: a retrospective observational study. Lancet Glob Health 4: e57–e68. S2214-109X(15)00215-6 [pii]; 10.1016/S2214-109X(15)00215-6 26718810 PMC5495601

[13] WHO (1995) *WHO* Physical status: the use and interpretation of anthropometry: 1–8 Nov 1993 geneva: WHO/TRS/854*;1995*.

[14] Burr SE, Hart J, Edwards T, Harding-Esch EM, Holland MJ, Mabey DC, Sillah A, Bailey RL (2014) Anthropometric indices of Gambian children after one or three annual rounds of mass drug administration with azithromycin for trachoma control. BMC Public Health 14: 1176 1471-2458-14-1176 [pii]; 10.1186/1471-2458-14-1176 25407464 PMC4251859

[15] Salman S, Davis TM, Page-Sharp M, Camara B, Oluwalana C, Bojang A, D'Alessandro U, Roca A (2015) Pharmacokinetics of Transfer of Azithromycin into the Breast Milk of African Mothers. Antimicrob Agents Chemother 60: 1592–1599. AAC.02668-15 [pii]; 10.1128/AAC.02668-15 26711756 PMC4775927

[16] Coles CL, Rahmathullah L, Kanungo R, Katz J, Sandiford D, Devi S, Thulasiraj RD, Tielsch JM (2012) Pneumococcal carriage at age 2 months is associated with growth deficits at age 6 months among infants in South India. J Nutr 142: 1088–1094. jn.111.156844 [pii]; 10.3945/jn.111.156844 22535764 PMC3349980

[17] Jasseh M, Webb EL, Jaffar S, Howie S, Townend J, Smith PG, Greenwood BM, Corrah T (2011) Reaching millennium development goal 4—the Gambia. Trop Med Int Health 16: 1314–1325. 10.1111/j.1365-3156.2011.02809.x 21707875

