# Supplementary figures and images for: Long-lasting effect of oral azithromycin taken by women during labour on infant nutrition: Follow-up cohort of a randomized clinical trial in western Gambia
Source: PLoS One. 2018 Oct 25;13(10):e0206348. doi: 10.1371/journal.pone.0206348 (PMC6201939; doi:10.1371/journal.pone.0206348)

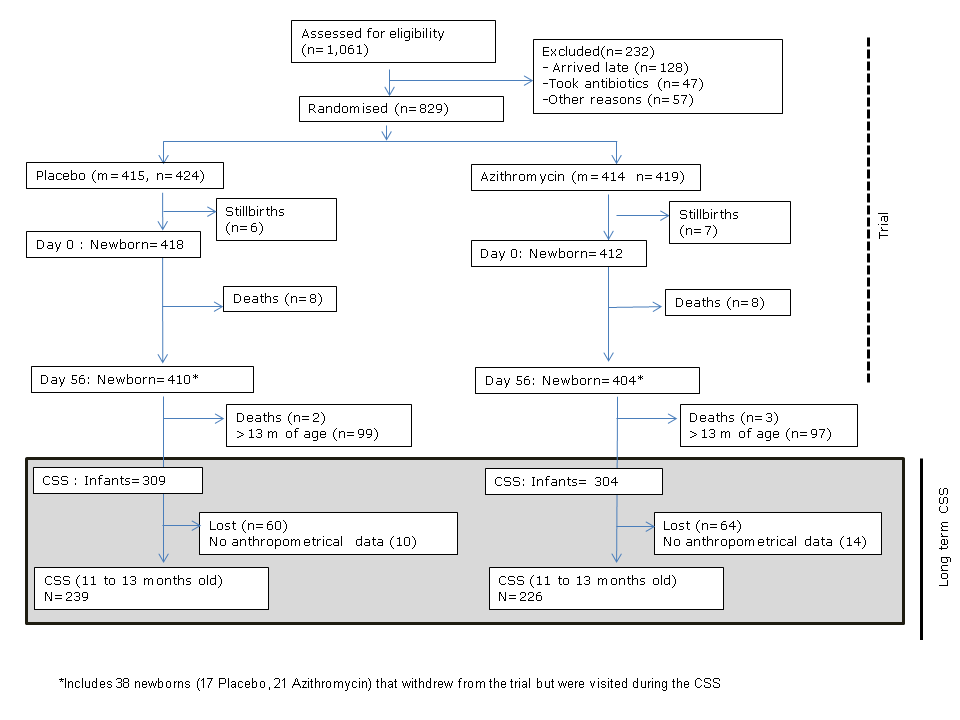

Supplement: S1 Fig — (TIF) [file pone.0206348.s002.tif]

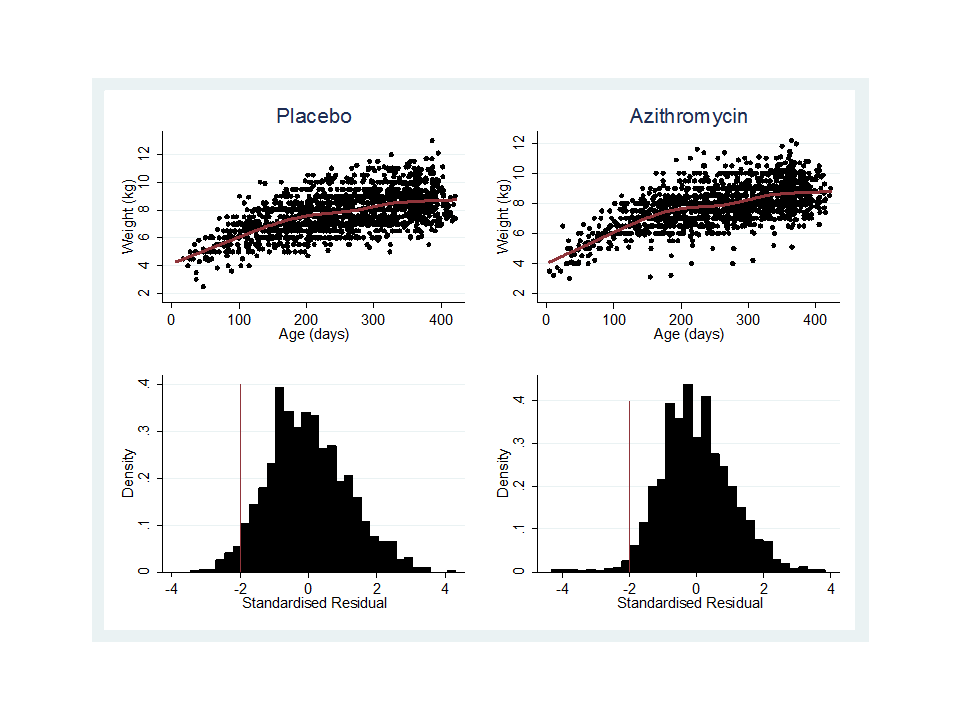

Supplement: S2 Fig — (TIF) [file pone.0206348.s003.tif]
